# A good career start can open doors: the plusses and minuses of an international graduate student program—a student’s perspective

**DOI:** 10.1007/s00430-020-00661-7

**Published:** 2020-02-13

**Authors:** Anchal Malik

**Affiliations:** Astbury Centre for Structural Molecular Biology, School of Biomedical Science, University of Leeds, Leeds, LS2 9JT England, UK

**Keywords:** Multidrug resistance, new diagnostic tools, good career start, International Ph.D. positions, ViBrANT, Multinational Ph.D. program, Increasing cases of bacterial diseases, Culture shock, Marie Skłodowska-Curie actions | Horizon 2020—European Commission, Settling into a new country, Multidisciplinary research

## Abstract

There are many Ph.D. programs from various funding agencies that provide excellent starts to a scientific career. Multinational Ph.D. positions attract students because they provide students with much-required exposure to the international scientific community at an early stage of the career. For this reason, multinational Ph.D. positions can be considered as a better career opportunity over Ph.D. positions confined to a single country. In addition, these multidisciplinary research programs connect different organizations to deal with the problems of global interest. One of these multi-disciplinary research programs is the viral and bacterial adhesion network training-innovative training network (ViBrANT). ViBrANT is a multifaceted platform that develops the required skillsets in young researchers and thereby also contributes to building a multidisciplinary research community. Is this the only parameter to be considered or are there other factors that can also stimulate one’s career development? In this perspective article, I will discuss the key reasons why I chose a multinational Ph.D. program along with the merits of being part of ViBrANT. I also discuss the challenges I faced while moving from India to the United Kingdom.

## Introduction

A good professional record is necessary to achieve higher positions in any industry. Science is no different. The number of published articles, their impact factors, good funding, national and international collaborations are some of the factors that are considered when a scientific career is being evaluated, and so one’s scientific credibility. The big question is “How to be a part of this race?”

To be a candidate for an international Ph.D. program requires having a good academic background and knowledge of the subject. Furthermore, brand names have a strong impact on our minds, even in the scientific community [1]. I have heard over and over again that if you are not a part of one of the high rated Universities [2] it is very hard to get into an international graduate program. Unfortunately, this statement is true and served as a cause for me to step out of my comfort zone and start applying for international Ph.D. programs.

The very first hurdle I faced was to look for appropriate opportunities in the web of advertised positions. It is very important to look for trusted websites: hence, I used the official web pages of the organizations. This is particularly for the universities or programs where I had no direct contact. The easiest is to search on the websites such as FindAPhD.com, the CCP4 Bulletin Board, jobs.ac.uk where people post various available opportunities in all sectors. Also, I paid major attention to the international ratings of the universities. My next move was to validate the feasibility of the project. There is no guarantee in science that everything will work, but reading articles published by the groups helped me to choose.

### Research driven by international collaborations

Is it enough to pursue higher education abroad or is there something that offers more benefits and all-round development? The answer lies in the increasing number of citations for multi-author papers [3]. Research over the past two decades is driven by collaboration among elite research groups across political borders [4]. This implies that multinational network projects are better than domestic projects. Not only are students attracted toward these multinational doctoral programs, but also both new as well as established scientists are highly motivated to write such collaborative grants. International research projects such as ITNs are not only confined to university-based research groups but also include both large and small companies. The merger of academia with industry is very important for the globalization of the research [5]. It also increases the possibility of translating basic research into accessible products of public interest.

I remember being introduced to one such multidisciplinary program—ViBrANT—when I contacted Professor Adrian Goldman regarding possible openings in his research group. This opportunity also motivated me to best present myself as the most suitable candidate for the position. I found the overall research focus of ViBrANT on combatting the problem of increasing cases of bacterial and viral diseases, predominantly in developing and underdeveloped countries, to be very appealing.

ViBrANT is a multidisciplinary training network project funded by “European Commission Horizon 2020 program”, that brings together nine academic institutions, one non-profit research institute, three small and medium-sized enterprises (SMEs), and one large enterprise to develop better and more cost-effective diagnostic kits and tools against deadly invasive bacterial diseases. ViBrANT not only allows me to work on a well-directed project but also provides multiple opportunities to learn the most advanced technique(s) often employed to address complex problems and to develop various skillsets, for example, scientific writing and public speaking which are also essential for a successful career. Networking is another major advantage offered by ViBrANT and other multinational research programs.

### A new age for research in disease diagnosis

While choosing a multinational Ph.D. program, I was also concerned that it met my research interests, which lie in combating the problem of emerging multidrug resistance in pathogens. The developing resistance in pathogens against current treatments has triggered the need for the development of improved and affordable care strategies.

One of the important characteristics of improved medical provisions is fast and accurate diagnosis of the disease. This is the first step for patient management and disease control but is still undervalued as compared to drugs and vaccines [6]. Improved diagnostic tools must identify the specific pathogen or at least provide clear discrimination between bacterial and viral infections [7]. These diagnostic tools should be fast, highly sensitive, cost-effective and user-friendly [7]. My inclination towards the objective is one of the reasons I applied for the ViBrANT Ph.D. program.

### Adjustment, adaptation, and acculturation

Is this everything that should be considered? What about the hidden hurdles of settling into a new country—even one that you know about from movies and almost as part of your own cultural background? We often neglect various other factors that may cause difficulties when we talk about a better career. The professional challenges are not the only ones that affect you. Other factors such as weather, lifestyle, language barriers and cultural differences can be challenging as well. For instance, the change in weather conditions can frustrate or relax your mind. I remember how hard it was for me to adjust to the cold and rainy weather of the United Kingdom.

The concept of culture shock and adaptation is not new to social biology. Traditional approaches from the literature have been reviewed to derive comprehensive approaches of culture learning, stress coping and social identification concerning the mental health of foreign students [8]. Psychological factors related to leaving your family and friends behind and intercultural adaptation such as learning the social skills of the society of settlement are the major factors governing the adjustment in a non-native land [8].

The difference in the language is one of the hurdles to deal with in the procedure of sociocultural adjustment. I knew that the language barrier could be problematic, but I never realized that the difference in accents between my Indian-English and all the varieties I hear in Leeds—received pronunciation (“BBC”), Yorkshire, Lancashire, Scots, German-English and so on—made me feel nervous at times. Adjusting to the new work environment and understanding the thought process of co-workers was as challenging as understanding the political statements. However, most of the times people were generous and helped me to understand them.

### Key steps for decision making

Another big challenge was to find a correlation between expectation and reality. I always kept in mind that things could be drastically different and peer pressure could be more than my expectation. These were the situations where I doubted my decision but, in these circumstances, I always weighed the benefits against the doubts: this helped me to find an answer. While talking to other people, I realized that these were common problems that are faced by almost everyone. This was something that faded away with time and I quickly became part of the group.

## Final thoughts

Selecting a competitive research question and research group for a doctoral degree play a critical role in securing positions in recognized firms. While opting for international Ph.D. programs is very motivating, it is also important to recognize the nature of the international Ph.D. program. With increasing numbers of Ph.D. graduates every year, the nature of training and the quality of publications you get become determining factors for early success in an academic career.

In this perspective article, I have discussed factors to be given priority while applying for international Ph.D. positions. In my opinion, evaluating the possible outcome of the project is as important as understanding the project itself. Such evaluation should not merely rely on the possible number of publications but also take other factors into consideration. These include the multidisciplinary nature of the proposed project, involvement of advanced technique(s) and possible collaborations.

I have also described the challenges associated with opting for an international multidisciplinary project for Ph.D. training. While meeting demands of such projects can be daunting, making oneself comfortable in a foreign country can be an equally challenging and time-consuming process. In my case, the motivation to learn and have a successful career has been the driving force to help me overcome my fears.
